# Supplementary material for: Active hepatocellular carcinoma is an independent risk factor of direct-acting antiviral treatment failure: A retrospective study with prospectively collected data
Source: PLoS One. 2019 Oct 3;14(10):e0222605. doi: 10.1371/journal.pone.0222605 (PMC6776434; doi:10.1371/journal.pone.0222605)
Supplement: S1 Table — (DOCX) [file pone.0222605.s002.docx]

Supplementary table 1. The comparison of SVR rates between patients with and without HCC stratified by genotype and treatment regimen

| Regimen | Genotype | HCC, N (%) | Non-HCC, N (%) | *P* |
| --- | --- | --- | --- | --- |
| GZR/EBR+RBV 12 weeks | 1b | 1/1 (100%) | 3/3 (100%) | - |
| GZR/EBR+RBV 12 weeks | 4 |  | 1/1 (100%) | - |
| GZR/EBR 12 weeks | 1a |  | 7/7 (100%) | - |
| GZR/EBR 12 weeks | 1b | 27/27 (100%) | 78/79 (98.7%) | 0.745 |
| 3D+RBV 12weeks in 11 patients,  24 weeks in 20 patients | 1a | 3/3 (100%) | 28/28 (100%) | - |
| 3D+RBV 12 weeks | 1b |  | 1/1 (100%) | - |
| 3D 12 weeks | 1b | 43/43 (100%) | 207/208 (99.5%) | 0.829 |
| 3D 12 weeks | 1b+6 |  | 1/1 (100%) | - |
| SOF+DCV+RBV 12 weeks | 2 | 15/18 (83.3%) | 31/31 (100%) | 0.044 |
| SOF+RBV 12 weeks | 2 | 46/50 (90.0%) | 207/215 (96.7%) | 0.162 |
| SOF/LDV +RBV 12 weeks | 1a |  | 7/7 (100%) | - |
| SOF/LDV +RBV 12 weeks | 6 | 1/1 (100%) | 5/5 (100%) | - |
| SOF/LDV +RBV 12 weeks | 1b | 9/9 (100%) | 25/25 (100%) | - |
| SOF/LDV 12 weeks | 1a | 1/1 (100%) | 15/15 (100%) | - |
| SOF/LDV 12 weeks | 1b | 17/17 (100%) | 53/53 (100%) | - |
| SOF/LDV 12 weeks | 6 | 8/8 (100%) | 25/26 (96.2%) | 0.765 |
| DCV/ASV 24 weeks | 1b | 17/18 (94.4%) | 75/75 (100%) | 0.200 |

*P*-value was estimated using one-sided Fisher’s exact test.

SVR, sustained virologic response; HCC, hepatocellular carcinoma; SOF, sofosbuvir; SOF/LDV, sofosbuvir plus ledipasvir; 3D, ritonavir-boosted paritaprevir, plus ombitasvir and dasabuvir; GZR/EBR, grazoprevir plus elbasvir; DCV, daclatasvir; ASV, Asunaprevir; RBV, ribavirin;
